# StarD7 deficiency hinders cell motility through p-ERK1/2/Cx43 reduction

**DOI:** 10.1371/journal.pone.0279912

**Published:** 2022-12-30

**Authors:** Mariano Cruz Del Puerto, María Laura Rojas, Ana Cristina Racca, Lucille Tihomirova Kourdova, Andrea Lis Miranda, Graciela Panzetta-Dutari, Susana Genti-Raimondi, Jésica Belén Flores-Martín

**Affiliations:** 1 Departamento de Bioquímica Clínica, Universidad Nacional de Córdoba, Facultad de Ciencias Químicas, Ciudad Universitaria, Córdoba, Argentina; 2 Consejo Nacional de Investigaciones Científicas y Tecnológicas (CONICET), Centro de Investigaciones en Bioquímica Clínica e Inmunología (CIBICI), Ciudad Universitaria, Córdoba, Argentina; University of Texas Medical Branch at Galveston, UNITED STATES

## Abstract

StarD7 belongs to START protein family involved in lipid traffic, metabolism, and signaling events. Its precursor, StarD7.I which is important for mitochondrial homeostasis, is processed to the StarD7.II isoform that lacks the mitochondrial targeting sequence and is mainly released to the cytosol. StarD7 knockdown interferes with cell migration by an unknown mechanism. Here, we demonstrate that StarD7 silencing decreased connexin 43 (Cx43), integrin β1, and p-ERK1/2 expression in the non-tumoral migratory HTR-8/SVneo cells. StarD7-deficient cells exhibited Golgi disruption and reduced competence to reorient the microtubule-organizing center. The migratory capacity of StarD7-silenced cells was reestablished when Cx43 level was resettled, while p-ERK1/2 expression remained low. Importantly, ectopic expression of the StarD7.II isoform not only restored cell migration but also ERK1/2, Cx43, and integrin β1 expression. Thus, StarD7 is implicated in cell migration through an ERK1/2/Cx43 dependent mechanism but independent of the StarD7.I function in the mitochondria.

## Introduction

Cell migration is crucial in a variety of physiological processes comprising embryonic development, immune surveillance, wound healing, and trophoblast invasion. Dysregulated migration is detected in many pathologic conditions such as inflammation, cancer, and also in pregnancy complications including pre-eclampsia, intrauterine growth restriction, placenta accreta spectrum, and gestational trophoblastic disease. Cell migration is a tightly regulated, multi-step, and highly dynamic process that involves a growing number of signaling pathways, cell surface receptors, transcription factors, cytoskeleton proteins, integrins, extracellular matrix (ECM), and ECM-associated proteins [1–4].

Connexin 43 (Cx43) is a member of a large family of integral membrane proteins that establish Gap Junction Intercellular Communication (GJIC) permitting the intercellular transference of ions, metabolites, and small signaling molecules [5]. Also, Cx43 forms hemichannels allowing the passage of molecules up to 1 kDa from the cytoplasm to the extracellular milieu and vice versa [6]. Besides the GJIC function, connexins have channel-independent functions affecting cell morphology through the cytoskeleton rearrangement controlling cell polarity and cell migration [2, 7, 8].

StarD7, a member of START lipid transfer protein family, was initially described in the trophoblast-derived JEG-3 cell line [9]. StarD7 is synthesized as a precursor protein named StarD7.I that contains a mitochondrial localization signal which is rapidly cleaved originating a shorter protein named StarD7.II mainly released to the cytosol [10, 11]. StarD7.I delivers phosphatidylcholine to the mitochondria [11–14]; its contribution to the trophoblast physiology [15] as well as in preserving endoplasmic reticulum (ER) and mitochondrial morphology and dynamics were established [11, 13, 14, 16, 17]. At present, a specific role of the abundant cytosolic StarD7.II isoform has not been reported. We have previously demonstrated that StarD7 knockdown reduces cell migration and Cx43 expression in JEG-3 cells [15, 18]; however the mechanism involved remains unexplored. Here, we used the migratory and non-tumoral HTR-8/SVneo cell line to uncover the mechanism and downstream targets. The study highlighted the role of the StarD7.II isoform in regulating polarized cell migration.

The results show that StarD7-dependent reduction in cell migration relates to ERK1/2/Cx43 expression. Furthermore, our findings indicate that StarD7 silencing causes a disruption of the Golgi apparatus and a deficiency in cell polarity. Through StarD7.I, StarD7.II, and Cx43 recovery assays, we demonstrate that the reduced cell migration in StarD7-deficient cells is dependent on Cx43 expression but independent of the well-known function of StarD7.I in the mitochondria. Altogether these results reveal a novel role for StarD7.II isoform in regulating cell migration.

## Materials and methods

### Antibodies

Antibodies against integrin α5 (sc-10729), integrin β1 (sc- 374429), p-FAK (Tyr 396, sc-11765-R), total FAK (C20, sc-558), p-ERK1/2 (sc-7383), ERK (K23, sc-94) were obtained from Santa Cruz. F(ab)2-goat anti-mouse IgG (H+L) cross-adsorbed secondary antibody Alexa Fluor 594 (A-11020) and F(ab)2- goat anti-rabbit IgG (H+L) cross-adsorbed secondary antibody Alexa Fluor 488 (A-11070), were from Thermofisher Scientific. Mouse monoclonal anti-α-tubulin (Clone B-5-1-2), anti-γ-tubulin (clone GTU-88) as well as rabbit polyclonal anti-Cx43 antibodies were from Sigma Chemical Co. Mouse monoclonal anti-GM130 (610823) antibody was from BD Transduction Laboratories; and mouse monoclonal anti-Golgin 97 (A-21270) antibody was from Molecular Probes. Anti-StarD7(C-terminal) antibody was produced in our laboratory [19]. IRDye 800CW donkey anti-rabbit IgG (P/N 926–32213), IRDye 800CW donkey anti-mouse IgG (P/N 926–32213) and IRDye 680RD donkey anti-mouse IgG (P/N 926–68072) antibodies were acquired from Li-Cor Biosciences.

### Cell culture and knockdown of endogenous StarD7

The HTR-8/SVneo immortalized trophoblast cell line was provided by C.H. Graham (Queen’s University, Ontario, Canada) [20]. Mycoplasma testing was regularly performed at the CIBICI Facility using PCR. Cells were cultured in DMEM/F-12 (Thermofisher) supplemented with 10% of fetal bovine serum (Internegocios S.A.) and 100 μg/μL penicillin, 100 μg/mL streptomycin (Thermofisher) at 37°C and 5% CO_2_. To knockdown endogenous StarD7 expression, cells were seeded in 6-well plates (1 x 10^5^ cells/well) and transfected 24 h later (confluence about 40%) with 50 nM of a specific StarD7 small interfering RNA (siRNA) (siD7) (sense: GGUAUAGUGUGGAUCAGGATT) using 4 μL of RNAimax (Thermofisher) per mL of OptiMEM as previously described [15, 16]. siD7 target sequence corresponds to position 1099–1117 of the StarD7 mRNA NM_020151.3. A scrambled siRNA (siC) was used as a negative siRNA control (Silencer Negative^™^) (Applied Biosystems/Ambion, Biosystems). Transfected cells were cultured for 72 h and medium refreshed each 24 h.

Alternatively, HTR-8/SVneo cells were stably transduced with a short hairpin RNA (shRNA) vector that targets the sequence CGGTTGGAAGAAATGTCAAAT located at position 711–731 of the StarD7 transcript. To this end, lentiviral particles were generated by transfecting HEK-293T cells with psPAX2, pMD2.G (Addgene) and pLKO.1 containing the shRNA sequence (shD7) against StarD7 (The RNAi Consortium clone ID TRCN0000151458, Sigma-Aldrich) or pLKO.1 empty (shC) vectors (SHC001, Sigma-Aldrich). Conditioned medium with viruses was collected after 72 h and used to transduce HTR-8/SVneo cells as described [13]. Cells were collected and StarD7 levels analyzed by Western blot and qRT-PCR. Stable cells were amplified and used for subsequent experiments with less than 5 passages.

### Transfection of StarD7-deficient cells (rescue experiments)

Cells stably silenced for StarD7 were transiently transfected with pLenti vectors expressing wild type StarD7.I (D7.I) or StarD7.II (D7.II) isoforms or pLenti empty (EV) constructed as described [13]. The Cx43 cDNA cloned in pLPCX-Cx43-IRES-GFP was kindly provided by Dr. Trond Aasen (Molecular Pathology, Hospital Universitari Vall d’Hebron—Institut de Recerca (VHIR), Universitat Autònoma de Barcelona, Passeig Vall d’Hebron 119–129, Barcelona, 08035, Spain). pLPCX-Cx43-IRES-GFP (pCx43-GFP) was digested with BglII to release the Cx43 insert and then religated to obtain pLPCX-IRES-GFP (EV-GFP) and both plasmids were used in transient transfections. The pCx43-GFP plasmid encodes Cx43 and GFP divided by IRES, thus both proteins are independently translated from one mRNA. Transfections were performed using Lipofectamine 3000 (Invitrogen) according to the manufacturer’s specifications. Cells were incubated for 24 h before proceeding with the experiments.

### Cell migration assay

HTR8/SVneo cell migration was determined by wound healing assays and performed in siC and siD7 as previously described [15]. To perform cell migration assay in stable shC and shD7 cells, they were seeded on twelve-well plates (1 × 10^5^ cells/well) and cultured to reach a confluence of 70%. Then, cells were transfected or not with EV-GFP, p-Cx43-GFP, EV, D7.I, or D7.II plasmids for 48 h, as indicated. Confluent monolayers were wounded and assessed under microscopy at 0 and 8 h after. The results were expressed as the percentage of the wound closure calculated as the difference between the remaining area at 8 h and the initial one at 0 h recorded in seven fields of duplicate wells from three independent experiments.

Analysis of the migration of individual cells was conducted employing an IN Cell Analyzer 2500 HS (High content analysis, GE Healthcare Life Sciences) in a time-lapse mode (one picture every 15 min interval over a total time of 120 min). Individual cell trajectories were manually tracked using Image J/ Fiji software. Only cells at the front line of migration were examined, using the border of the cell as the reference mark for cell movement. Movies are presented in the S1–S3 Movies.

### RNA extraction and quantitative reverse transcription-polymerase chain reaction (qRT-PCR)

Total RNA was purified with Trizol (Thermofisher), following the manufacturer’s instructions. Single-stranded cDNAs were synthesized and qRT-PCR was carried out with SYBR Green PCR Master Mix (Applied Biosystems) using the primers for StarD7 and cyclophilin A as previously described [13]. The transcript levels were normalized to those of cyclophilin A and the relative expression levels were quantified as described [13]. Amplification efficiency for each set of primers was near 98%. No amplification was detected in PCR reactions using water instead of template.

### SDS-PAGE and Western blotting

Western blot was performed as previously reported [15]. Briefly, protein extracts were loaded onto 10% SDS-PAGE gels, migrated and electrotransferred to nitrocellulose (Amersham Bioscience). After blocking, the membranes were incubated with the following antibodies: anti-StarD7(C-terminal) (0.5 μg/mL), anti-α-tubulin (1:3000), anti-Cx43 (1:2000), anti-integrin α5 (1:300), anti-pFAK (1:500), anti-total FAK (1:500), anti-pERK1/2 (1:2500), anti-total ERK1/2 (1:2500) or anti-integrin β1 (1:300). After washing, the blots were incubated with IRDye 800CW donkey anti-rabbit, IRDye 800CW donkey anti-mouse, or IRDye 680RD donkey anti-mouse IgG antibodies (1:15000). The proteins were visualized and measured with the Odyssey Infrared Imaging System (LI-COR, Inc., Lincoln, NE, USA). The densitometric quantification of the indicated protein levels in siD7 or shD7 cells were normalized to α-tubulin and expressed relative to the normalized protein levels in the siC or shC cells, respectively; while the p/FAK/FAK and p-ERK/ERK ratio in shD7 cells are expressed relative to corresponding ratio in shC cells.

### Immunofluorescence

HTR-8/SVneo cells (5 x 10^5^ cells/well) were seeded on coverslips in 6-well plates and incubated in supplemented DMEM/F-12 at 37°C and 5% CO_2_. Cells were cultured to reach a confluence of 70% and then, immunofluorescence was performed as previously described [15] using the following antibodies: anti-GM130 (1:200), anti-Golgin 97 (1:100), or anti-Cx43 (1:50). After washing, cells were incubated with Alexa Fluor 594 conjugated goat anti-mouse IgG or Alexa Fluor 488 conjugated goat anti-rabbit IgG. Hoechst 33342 dye was used to counterstain the nuclei. Slides were mounted using Mowiol 4–88 reagent (Sigma) and observed in a Fluorescence microscope DMI 8 (LEICA, Wetzlar, Germany) or a Confocal Olympus FLuoView FV 300 microscope (Olympus Latin America, Miami, FL), as indicated.

For microtubule-organizing center (MTOC) orientation analysis, a wound was conducted on confluent StarD7-silenced or control cell monolayers and 6 h later the cells were fixed and stained for γ-tubulin (1:2000) and Hoechst 33342 as described above. Cells with MTOCs located in the 120-degree sector facing the leading edge were considered oriented and cells with MTOCs positioned outside this sector were recorded as not oriented as described [21]. Cells located at the border of the wound were examined, and percentages were obtained from 350 cells per condition and assay.

Cx43 mean fluorescent intensity (MFI) was quantified using Image J/Fiji software over 50 cells per condition from images obtained at 630x in Fluorescence microscope DMI 8 (LEICA, Wetzlar, Germany).

### ROS and mitochondrial membrane potential (Δψm) detection

Mitochondrial ROS levels in live cells were detected using the fluorogenic probe MitoSOX Red in media without phenol red (Invitrogen) as described [13]. JC-1 iodide dye (5,5′,6,6′-tetrachloro-1,1′,3,3′-tetraethylbenzimidazolylcarbocyanine, iodide) staining was used to detect Δψm. Cells were washed with PBS, incubated with JC-1 iodide dye (2μM) for 20 min at 37°C in the dark and finally collected to analyze by flow cytometry (FACSCanto II, Becton-Dickinson) as described [13].

### Data analysis

All quantitative data were expressed as mean ± SEM of independent experiments and analyzed using the one sample t-test, Student’s t-test, or one-way ANOVA as indicated. Dunn’s post-test was performed for multiple comparisons of independent samples. Significance was taken as p< 0.05.

## Results

### StarD7 deficiency leads to reduced expression of Cx43 and other proteins involved in cell migration

**Fig 1:**
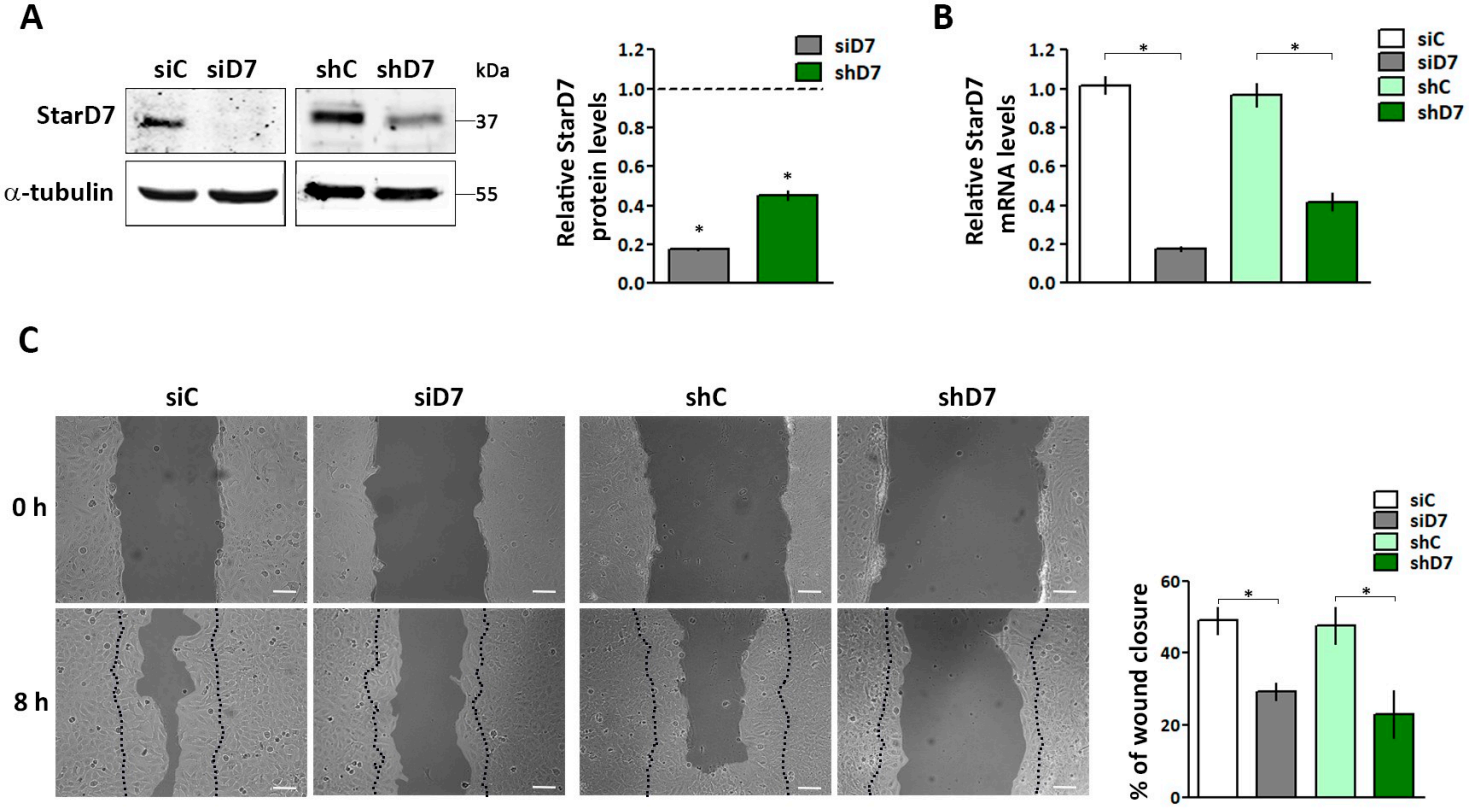
StarD7 protein levels and cell migration in StarD7 knockdown HTR-8/SVneo cells. (A) Representative Western blots of cell lysates obtained from siC, siD7, shC, or shD7 cells. The bar graph represents the densitometric quantification of StarD7 protein levels in siD7 or shD7 cells, normalized to α-tubulin of five independent experiments relative to the normalized protein levels in the corresponding controls (siC or shC). Statistical significance *vs*. the control defined as 1 was evaluated by one-sample t-test. (*p<0.01). (B) Relative StarD7 mRNA expression was determined by qRT-PCR. The values represent the mean ± SEM of at least three independent experiments performed by triplicate (siD7 *vs* siC; shD7 *vs* shC, *p< 0.01, Student’s t-test). (C) Representative experiments of wound healing assays performed in siD7, siC, shD7, or shC cells. Scale bar = 30 μm (100x). The bar graph represents the percentage of the wound closure at 8 h after scratch from three independent experiments (siD7 *vs* siC; shD7 *vs* shC, mean ± SEM, *p< 0.01, Student’s t-test).

We have previously documented that StarD7 silencing with a siRNA inhibits cell migration in JEG-3 cells [15]. Herein, we studied the underlying mechanism using the non-tumoral HTR-8/SVneo cell line characterized by a migratory phenotype. StarD7 siRNA (siD7) and shRNA (shD7) were used to deplete StarD7 levels. We verified that in both experimental conditions StarD7 protein levels were significantly decreased by > 90% and 60%, respectively (Fig 1A). Also, reduced StarD7 mRNA expression was determined by qRT-PCR (Fig 1B). As reported in JEG-3 cells, transient transfection with siD7 reduced HTR-8/SVneo cell migration evaluated by wound healing assays. In addition, cell migration was also reduced in stable StarD7-silenced HTR-8/SVneo cells (Fig 1C).

**Fig 2:**
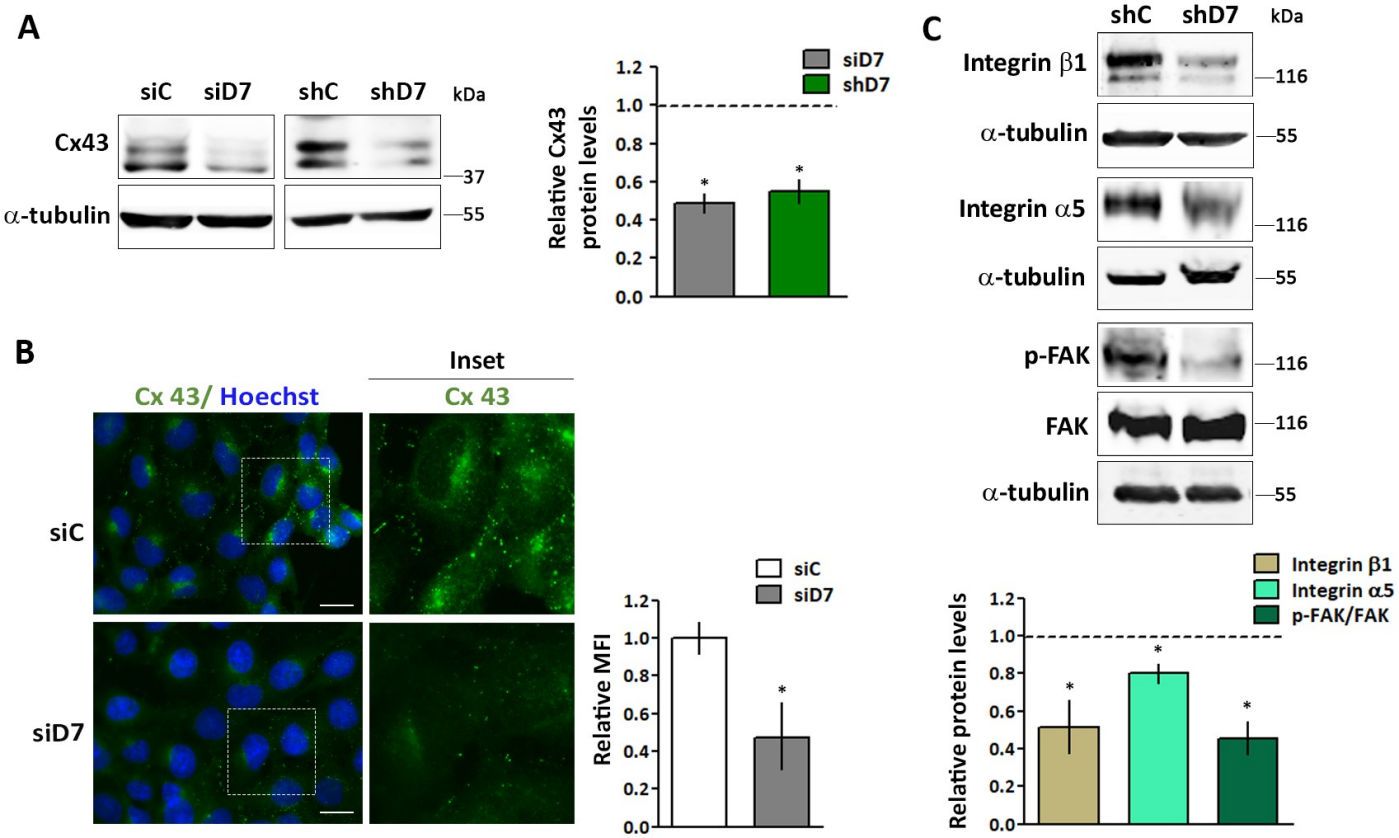
StarD7 silencing leads to a reduction in Cx43, integrin β1, integrin α5, and p-FAK expression. (A) Representative Western blots of Cx43 and α-tubulin from protein extracts of siC, siD7, shC, and shD7 cells. Bar graph represents the densitometric quantification of Cx43 protein levels in siD7 and shD7 cells normalized to α-tubulin of five independent experiments relative to the normalized protein levels in the corresponding controls (siC or shC). Statistical significance *vs*. the control defined as 1 was evaluated by one-sample t-test (Mean ± SEM, *p<0.01). (B) Representative microscopy of merged images demonstrating Cx43 expression (green) and nuclei labeled with Hoechst (blue) in siC- (top panel) and in siD7-treated cells (bottom panel). The boxed regions are enlarged in the right panels. Scale bar = 10 μm (630x). The bar graph represents Cx43 MFI in siC and siD7 cells relative to siC cells defined as 1 analyzed by Image J/Fiji software. (C) Representative Western blots of the indicated proteins from extracts of shC and shD7 cells. Bar graph represents the densitometric quantification of the integrin β1 and integrin α5 protein levels in shD7 cells normalized to α-tubulin relative to the normalized protein levels in the shC cells; and p-FAK/FAK ratio in shD7 cells relative to the corresponding ratio in shC cells of five independent experiments. Statistical significance *vs*. the control defined as 1 was evaluated by one-sample t-test (mean ± SEM, *p<0.01).

As mentioned, earlier exploratory data suggest that Cx43 mRNA is largely downregulated in JEG-3 StarD7-silenced cells [18]. In addition, numerous reports have linked Cx43 down-regulation with reduced cell motility and invasion [2, 7, 8]. Therefore, we explored Cx43 expression in HTR8/SVneo cells where StarD7 expression was decreased by siRNA or shRNA. We found that StarD7 silencing led to a reduction in Cx43 protein levels in both experimental conditions (Fig 2A). Three bands were detected on Western blots indicating phosphorylation of Cx43, as reported [22, 23]. Accordingly, abundant punctuate Cx43 immunostaining was observed in control samples while a significant MFI reduction was noticed in StarD7-silenced cells, confirming the data obtained from Western blot analysis (Fig 2B). Considering that Cx43 interacts with ECM and ECM-associated proteins connected to the migration process [7, 24–27], we assessed whether the reduced cell migration induced by the loss of StarD7 also affects the expression of well-known key proteins connected with this process such as integrin β1, integrin α5, and p-FAK. Western blot assays revealed that the amount of all the assayed proteins was reduced in shD7 cells compared to shC cells (Fig 2C).

### The decrease in HTR-8/SVneo cell migration was accompanied with a reduction in the ERK pathway activation

**Fig 3:**
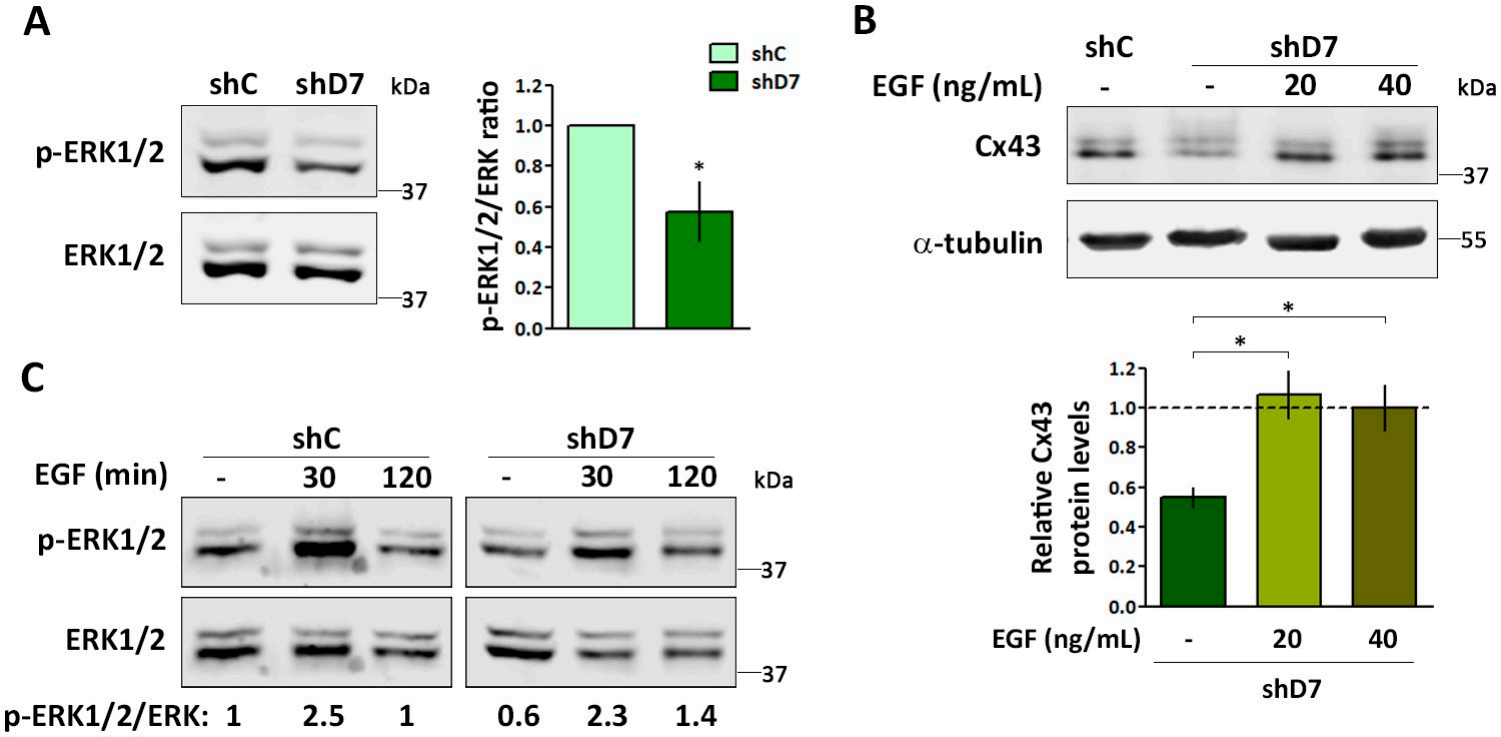
The decrease in HTR-8/SVneo cell migration was accompanied with a reduction in the ERK1/2 activation pathway. (A) A representative Western blot assay of p-ERK1/2 and total ERK from protein extracts of shC and shD7 cells. The bar graph represents the densitometric quantification of p-ERK1/2 protein normalized to total ERK expression in shD7 relative to the corresponding normalized levels in shC cells defined as one. Values are mean ± SEM of three independent experiments (*p< 0.05, one sample t-test). (B) A representative Western blot assay of Cx43 from protein extracts of shD7 cells treated or not with EGF 20 or 40 ng/mL for 8 hours. Bar graph represents the densitometric quantification of Cx43 protein levels in shD7 cells normalized to α-tubulin of three independent experiments relative to the normalized protein levels in shC cells defined as 1 (mean ± SEM, *p< 0.05, one-way ANOVA). (C) A representative Western blot assay of three independent experiments of p-ERK1/2 and total ERK from protein extracts of shC and shD7 cells treated or not with 15 ng/mL EGF during 30 and 120 min. The p-ERK/ERK ratio in the EGF-treated or not shD7 and EFG-treated shC cells relative to untreated shC cells is indicated below.

Numerous evidences reveal that ERK pathways are required to promote cell motility [28, 29]. In addition, several reports indicate that Cx43 is involved in regulating ERK1/2 signaling [30–34]. Thus, we explored the expression of p-ERK1/2 in StarD7-silenced cells. As illustrated in Fig 3, a significant decrease in p-ERK1/2 level was detected in StarD7-knockdown cells (Fig 3A). Interestingly, when shD7 cells were treated with 20 or 40 ng/mL of EGF during 8 h, the amount of Cx43 was increased in comparison with non-treated cells, indicating that the ERK1/2 pathway is upstream of Cx43 expression (Fig 3B). Additionally, p-ERK1/2 levels also increased following the treatment with 15 ng/mL of EGF in shD7 as in shC cells indicating that this signaling pathway is conserved in shD7 cells (Fig 3C).

### StarD7 silencing causes a deficiency in cell polarity and a disruption of the Golgi apparatus

**Fig 4:**
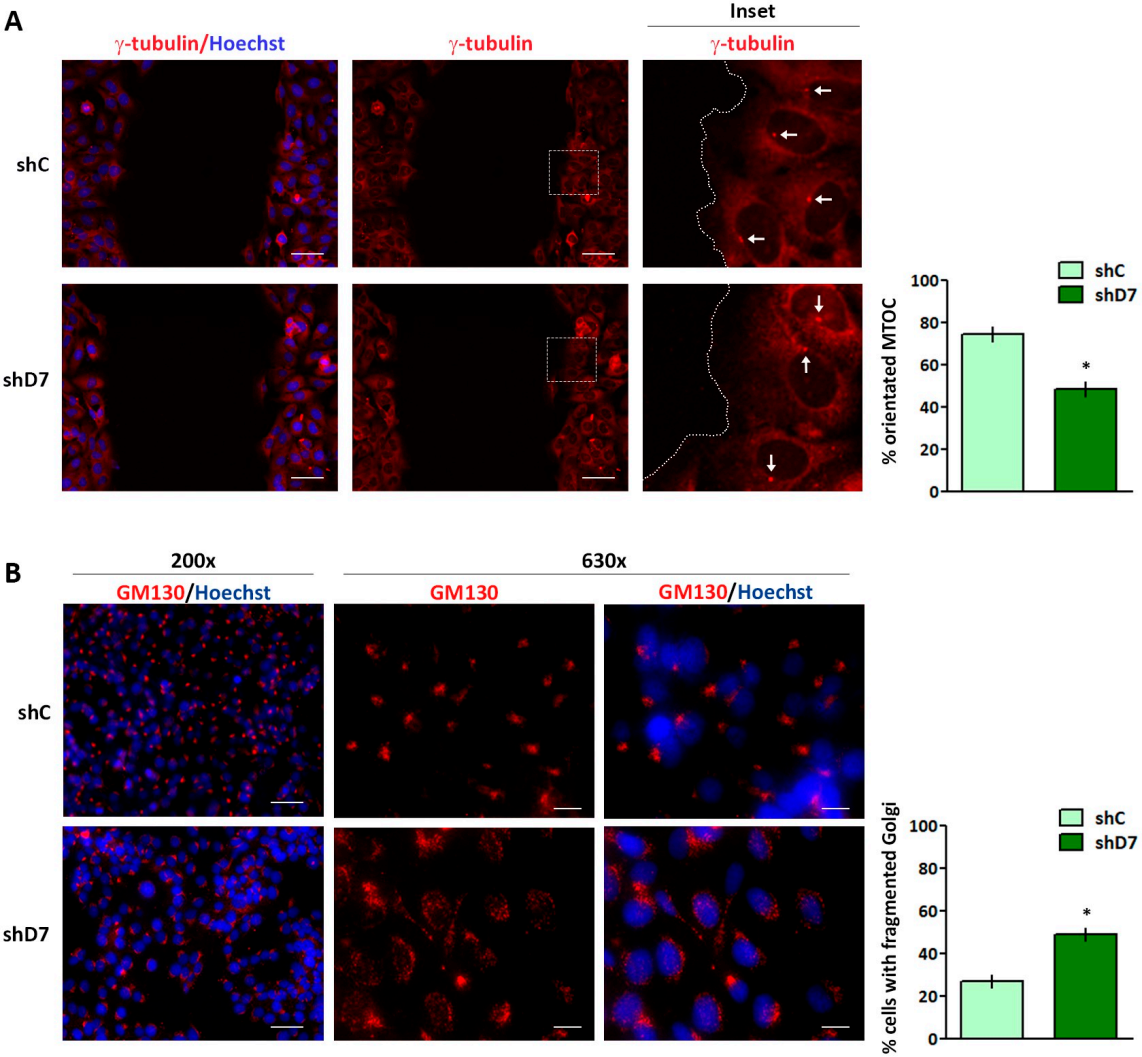
shRNA-mediated StarD7 knockdown impairs MTOC reorientation and disrupts the Golgi apparatus. (A) The monolayer of shC or shD7 cells was wounded and 6 hours later cells were immunostained with anti-γ-tubulin (red) to detect MTOC (arrows). The boxed regions are enlarged in the right panels. The nuclei were labeled with Hoechst (blue), merged images are shown on the left. The images were recorded by fluorescence microscopy and white lines indicate the wound edge. Scale bar = 30 μm (200x). Bar graph depicts the percentage of shC and shD7 cells exhibiting reoriented MTOC after wounded from two independent experiments (mean ± SEM, *p< 0.01, Student’s t-test). (B) Golgi apparatus morphology was visualized by fluorescence microscopy in shC and shD7 cells using anti-GM130 antibody (red). Images at 200x (on the left) and at 630x (on the right) are shown. The nuclei were labeled with Hoechst (blue). Scale bars = 30 μm (200x) and 10 μm (630x). Bar graph depicts the percentage of shC and shD7 cells exhibiting disrupted Golgi respect to total counted cells from two independent experiments (mean ± SEM, *p< 0.05, Student’s t-test).

Considering that Cx43 deficiency causes a failure of the MTOC to reorient with the direction of wound closure [21, 35], we investigated the location of MTOC during polarized cell migration. To this end, wound healing assays were carried out in StarD7-silenced and control cells and the MTOC was visualized through γ-tubulin immunofluorescence staining. In control cells the MTOC was principally located towards the direction of cell migration, as expected. In contrast, in the shD7 and siD7 conditions, the percentage of cells with oriented MTOC was reduced indicating a deficiency in cell polarity (Fig 4A and S1A Fig).

Taking into account that the directional cellular locomotion depends on the integrity of the Golgi apparatus [36], we explored Golgi morphology in StarD7-silenced cells by immunofluorescence staining of GM130, a cis-Golgi resident protein. As expected, GM130 staining was localized in the Golgi complex showing a condensed and perinuclear pattern in control cells (siC or shC) whereas in StarD7-silenced cells, GM130 signal appeared as a punctuated dispersed pattern indicative of Golgi disruption (Fig 4B and S1B Fig). Quantification showed that 49.0 ± 3.0% of shD7 cells exhibited a disrupted Golgi phenotype compared to only 27.0 ± 3.0% of shC cells (Fig 4B). Similar results were observed in transiently silenced StarD7 cells (S1B Fig). Golgi disruption induced by StarD7 downregulation was also confirmed by immunofluorescence staining of the trans-Golgi network protein Golgin 97 in siD7 cells (S1C Fig).

### Exogenous Cx43 restores cell migration but not ERK1/2 phosphorylation in StarD7-deficient cells

**Fig 5:**
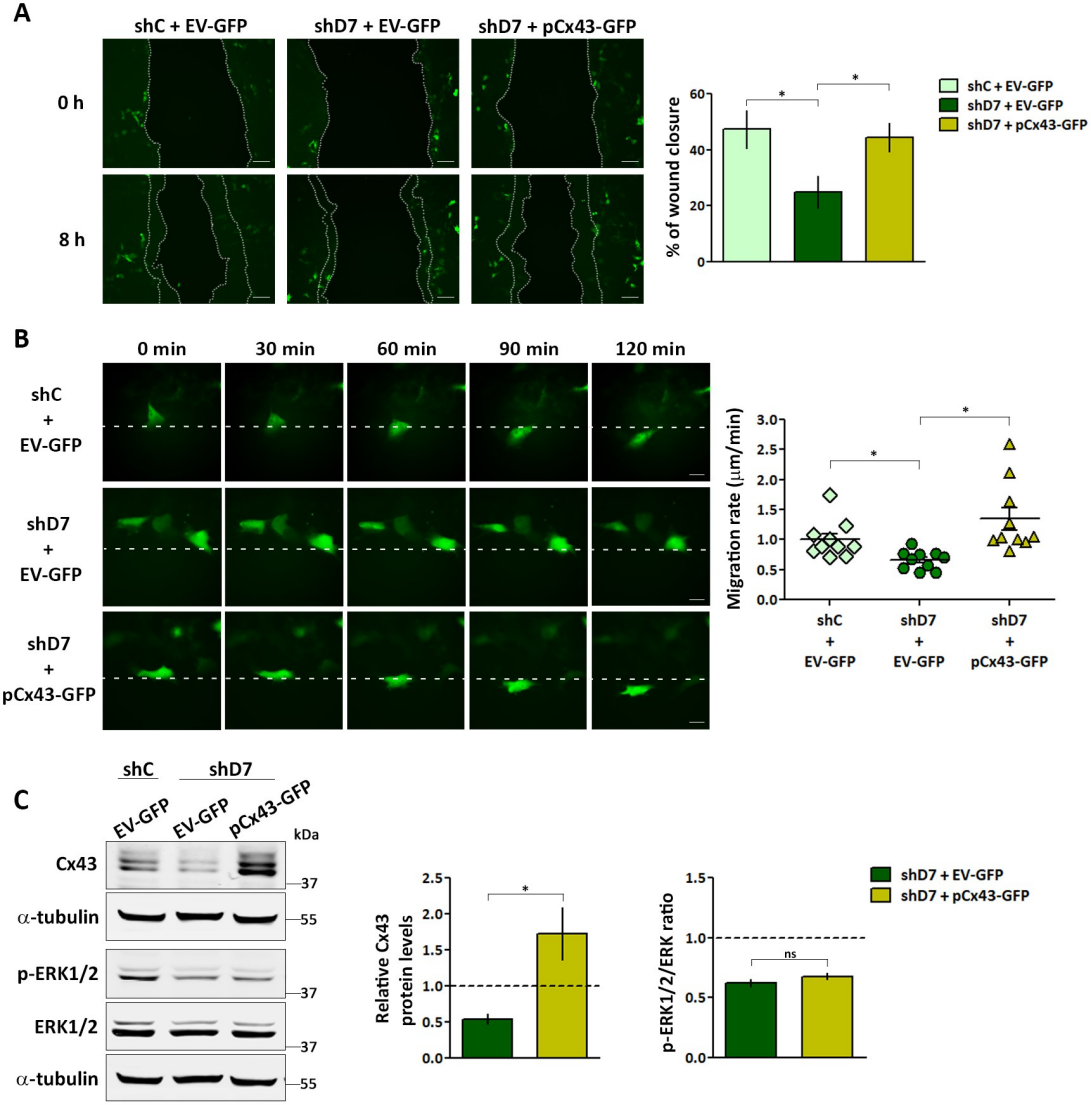
Exogenous Cx43 restores cell migration but not ERK1/2 phosphorylation in StarD7-deficient cells. (A) Representative experiments of wound healing assays performed in shC cells transfected with EV-GFP or shD7 cells transfected with EV-GFP or pCx43-GFP plasmids and visualized by fluorescence microscopy, as indicated. Bar = 30 μm (100x). The bar graph represents the percentage of the wound closure at 8 h after scratch from three independent experiments (mean ± SEM, *p< 0.01, one-way ANOVA). (B) Migration of individual cells was monitored in shC and shD7 cells transfected with EV-GFP, or shD7 cells transfected with pCx43-GFP, as indicated, using real-time imaging in 10 cells; the scatter plot shows the migration rate as mean ± SEM (*p< 0.01, one-way ANOVA). Scale bar, 10 μm (630x). (C) A representative Western blot assay of Cx43, p-ERK1/2 and total ERK1/2 from protein extracts of shC cells transfected with EV-GFP and shD7 cells transfected with EV-GFP or pCx43-GFP plasmids. Bar graphs represent the densitometric quantification of Cx43 protein levels in shD7 cells normalized to α-tubulin relative to the normalized protein levels in shC cells (left graph) and p-ERK1/2/ERK ratio in shD7 cells relative to the corresponding ratio levels in shC cells (right graph) defined as 1 from three independent experiments (mean ± SEM, *p< 0.01, Student’s t-test).

To confirm that the altered phenotype observed in StarD7-deficient cells was due to the Cx43 reduction, we restored Cx43 levels by exogenous addition. To this end, we transfected shD7 cells with the pCx43-GFP or EV-GFP plasmids and analyzed the capacity of cells to migrate in a polarized direction in comparison with shC cells transfected with EV-GFP. As shown in Fig 5A, the percentage of the wound closure area in shD7 cells transfected with pCx43-GFP was higher than in shD7 cells transfected with EV-GFP and similar to that observed in the control, thus the exogenous addition of Cx43 protein restores cell migration capacity. Migration of individual cells monitored by real-time imaging of GFP-fluorescent cells confirmed cell migration recovery of StarD7-deficient cells when Cx43 levels were increased, further highlighting that StarD7 modulates cell migration through Cx43 (Fig 5B and S1–S3 Movies). Interestingly, the p-ERK1/2 levels in shD7 cells were not modified by the addition of exogenous Cx43 compared to shD7 cells transfected with EV-GFP (Fig 5C), further supporting that the ERK1/2 pathway is upstream of Cx43 expression since, as mentioned above, ERK1/2 activation was able to increase Cx43 expression in shD7 cells (Fig 3B). Collectively these data suggest that StarD7 regulates polarized cell migration through Cx43 and suggest a mechanism that involves the p-ERK1/2/Cx43 signaling pathway.

### Exogenous StarD7.I and StarD7.II expression restores Cx43, integrin β1, p-ERK expression and cell migration

**Fig 6:**
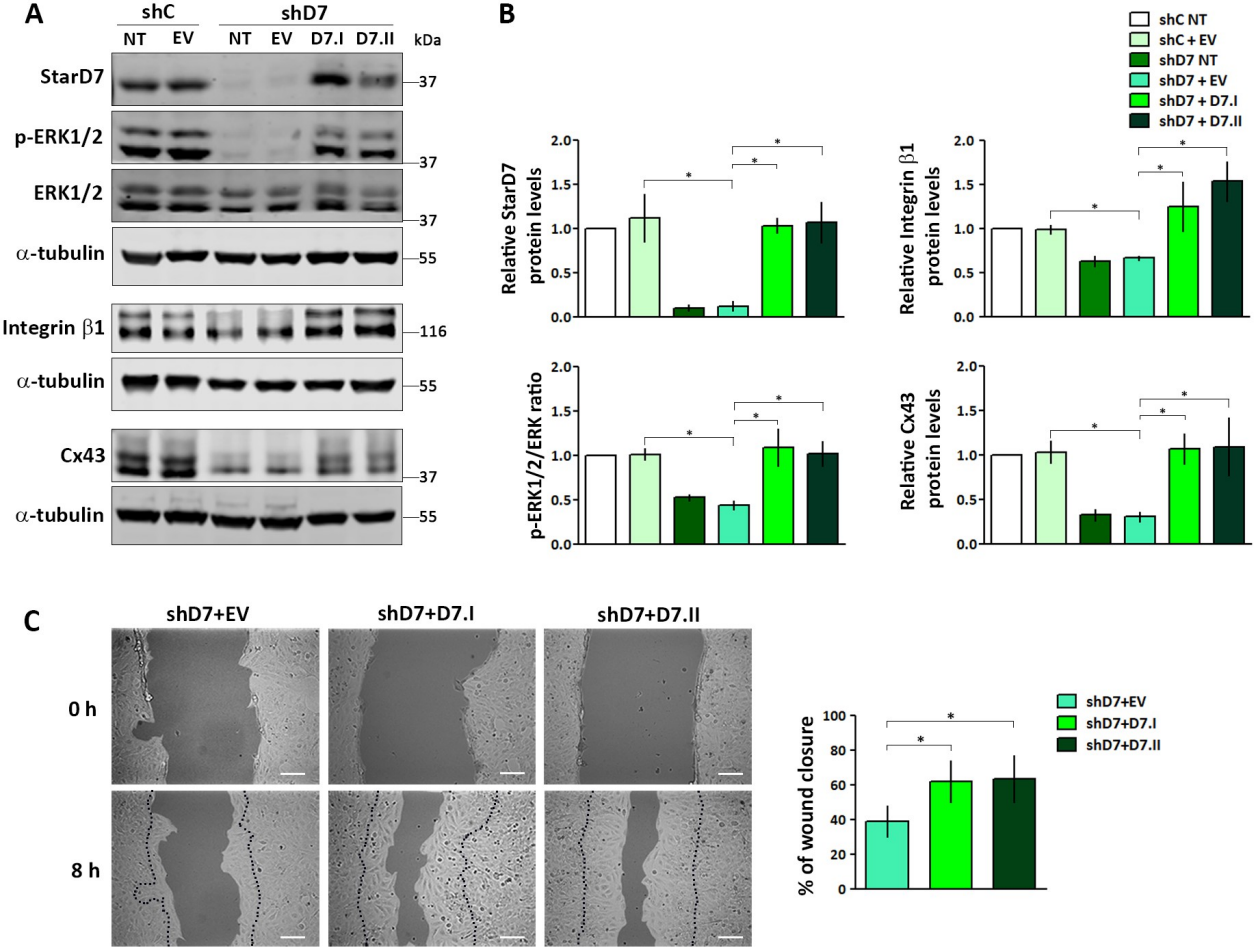
Exogenous StarD7.I and StarD7.II expression restores Cx43, integrin β1, p-ERK expression and cell migration. (A) Representative Western blot assays of StarD7, Cx43, integrin β1, p-ERK1/2, total ERK, and α-tubulin expression from protein extracts of shC cells transfected with pLenti empty vector (EV) or not (NT) as well as shD7 cells transfected with pLenti vectors encoding StarD7.I (D7.I) or StarD7.II (D7.II) isoforms, EV, or not transfected (NT). (B) The bar graphs represent the densitometric quantification of the StarD7, integrin β1, and Cx43 proteins in transfected-shC and -shD7 cells normalized to α-tubulin relative to the normalized protein levels in the non-transfected shC cells and p-ERK/ERK ratio in transfected-shC and -shD7 cells relative to non-transfected shC cells defined as 1. Values are mean ± SEM of three independent experiments (*p< 0.01, one-way ANOVA). (C) Representative experiment of wound healing assays performed in shD7 cells transfected with D7.I, D7.II, or EV. The bar graph represents the percentage of the wound closure at 8 h after scratch from three independent experiments (mean ± SEM, *p< 0.01, one-way ANOVA). Scale bar = 30 μm (100x).

In order to confirm that the reduced expression of Cx43 and of the other reported proteins, as well as cell migration impairment is a consequence of StarD7 deficiency, we performed rescue experiments. To this end, StarD7 knockdown cells were transfected with plasmids encoding the StarD7.I or StarD7.II isoform (D7.I and D7.II, respectively) or with the EV control. Transfection with either D7.I or D7.II plasmids restored mature StarD7 expression to a level like that observed in non-silenced shC cells, as demonstrated by western blot (Fig 6A). The detection of the mature StarD7 isoform after transfection with the D7.I plasmid was as expected, since the StarD7.I protein has an N-terminal mitochondrial localization signal which is rapidly processed in the mitochondria originating the mature StarD7.II isoform [11, 13, 14, 16, 17]. Exogenous expression of StarD7.I reestablished Cx43, integrin β1, and p-ERK1/2 protein to levels comparable to those observed in shC cells, while the expression of these proteins remained unmodified in cells transfected with the EV control ruling out a nonspecific effect due to the vector or the transfection protocol (Fig 6A and 6B). Remarkably, the exogenous expression of the StarD7.II isoform also restored protein levels to those observed in shC cells (Fig 6A and 6B). These results confirm that reduced Cx43, integrin β1, and p-ERK1/2 protein levels are due to StarD7 silencing. Moreover, cell migration was also recovered in StarD7-silenced cells transfected with either isoform, further confirming that the altered phenotypes observed in knockdown cells are a consequence of StarD7 deficiency (Fig 6C). Interestingly, since exogenous expression of StarD7.II restores cell phenotype, and this isoform is lacking the mitochondrial targeting sequence [11, 13, 14, 16, 17], present results suggest StarD7 role in cell migration is independent of the function of StarD7.I in the mitochondria.

To rule out that the reduction in cell migration is dependent on the well-documented role of StarD7.I in supporting mitochondrial activity [11, 13, 14, 16, 17], we measured mitochondrial ROS levels and the Δψm, as indicator of mitochondrial activity. Δψm and mitochondrial ROS levels were not modified in shD7 cells as compared to the control (S2 Fig).

## Discussion

This study provides an understanding of the role of StarD7 in HTR-8/SVneo cell migration and uncovers the main targets mediating StarD7 effects on its motility behavior. Herein, through siRNA/shRNA silencing, and protein rescue experiments, we show that StarD7 deficiency leads to a reduction in Cx43 expression resulting in defects in polarized cell migration.

Cell migration is a complex, multi-step, and highly dynamic process that involves a growing number of signaling pathways, cell surface receptors, transcription factors, cytoskeleton proteins, integrins, ECM, and ECM-associated proteins, in addition to Gap junction proteins [1–4]. Interestingly, a number of evidences clearly indicate that Cx43 interacts with ECM and ECM-associated proteins connected to the migration process [7, 24–27]. Present results show that StarD7-dependent Cx43 downregulation was associated with a decreased integrin α5, integrin β1, and p-FAK protein levels. In addition, StarD7 knockdown decreased ERK1/2 phosphorylation. Numerous evidences reveal that ERK pathways play an important role in promoting cell motility [28, 29]. Although, several studies indicate that ERK1/2 signaling pathway regulates Cx43 [37–41]; other reports involves Cx43 in the regulation of ERK1/2 signaling pathway [30–33]. Our data demonstrate that ERK1/2 phosphorylation was recovered by the addition of either StarD7 isoforms but not by exogenous Cx43, indicating that in this cell model ERK1/2 phosphorylation is upstream of Cx43. Remarkably, the addition of EGF to shD7 cells not only increased ERK1/2 phosphorylation, but also Cx43 levels reinforcing the idea that StarD7 silencing reduces Cx43 expression via ERK1/2 signaling pathway resulting in reduced cell motility. In this regard, Hino et al. have demonstrated the importance of the periodic ERK activation in the form of waves for collective cell migration [42]. Although it was not evaluated, it is possible that increased Cx43-mediated cell communication contributes with ERK waves transferring signaling molecules that enhance polarized cell migration. Alternatively, ERK-induced Cx43 expression might enhance cell migration through its channel-independent function known to control cytoskeleton morphology, directional migration, and organelle polarity, acting as a signaling molecule [2, 7, 8, 21, 43]. In this sense, Zhao et al. reported that betacellulin, a member of the EGF family, induces Cx43 expression through the activation of the MEK-ERK signaling pathway promoting ovarian cancer cell migration, and this effect is likely gap junction-independent [37]. In addition, we cannot discard the participation of Cx43 or other membrane channel proteins, such as Pannexins, that contribute to ERK signaling during migration through the release or uptake of ATP [44, 45].

It is well-known that directional cell migration is determined by the polarized distribution of protrusions at the cell periphery; and that cell polarity, which is a precondition for directed cell migration, is altered after Golgi apparatus disruption by pharmacological inhibition or by knocking-down structural proteins [46, 47]. Here, we show that StarD7 suppression leads to decreased Cx43 protein levels, Golgi apparatus disruption and a reduced cell capability to reorient the MTOC in a polarized migration. Our results are in line with the defects in cell polarity observed in mouse embryonic fibroblasts [21] and mouse epicardial cells [35] where a decrease in Cx43 levels, breakdown in the localization of the Golgi apparatus and disoriented MTOC next to the wound closure were detected. Importantly, the exogenous expression of Cx43 in StarD7-depleted cells was able to restore the motility cell behavior indicating that reduced StarD7 levels impair polarized cell migration, at least in part, through Cx43.

Furthermore, re-establishing the amount of StarD7.I or StarD7.II protein substantially restored Cx43, integrin β1 and p-ERK1/2 levels to those observed in the non-silenced cells accompanied by a recovery of cell migration, confirming the specificity of the effect. In addition, our data reveal that the migratory phenotype observed in StarD7-deficient cells is not associated with an important mitochondrial bioenergetic defect since mitochondrial membrane potential as well as mitochondrial ROS levels were not altered. More significantly, they indicate that the reduction in cell migration, which is modulated by Cx43 expression level, is due to a decreased StarD7.II isoform level, and is independent on the role of StarD7.I in mitochondrial morphology and dynamics [11, 13, 14, 16, 17]. Although we cannot rule out that StarD7 deficiency engages cell migration by inflicting other forms of mitochondrial damage, our data favor a model in which StarD7.II isoform deficiency followed by Cx43 reduction is responsible for the cell migration defects observed.

It has been reported that StarD7 haploinsufficient mice present alterations in lung epithelial barrier function with an enhanced allergic response, spontaneous atopic dermatitis, and decreased amount of the key tight junction protein zonula occludens-1 (ZO-1) [14, 48]. Interestingly, Erythrokeratodermia Variabilis et Progressiva [49] and severe dermatitis, multiple allergies, and metabolic wasting syndrome were reported to be caused by mis-localized Cx43 or enhanced Cx43 turnover [50]. Considering that ZO-1 is a well-known protein able to interact with Cx43 [51], it is attractive to hypothesize that the alteration in the epithelial barrier functionality observed by Yang et al. [48] in StarD7-knockdown airway cells could be a consequence of Cx43 reduction. Moreover, Yang et al. reported that the majority of StarD7^−/−^ mice died between embryonic day E10 and E11 most likely due to the disruption of cardiovascular development [48]. Similarly, Cx43 knockout mice die soon after birth as a consequence of cardiac defects connected with pulmonary outflow obstruction and coronary abnormalities [35, 52, 53]. Therefore, we speculate that the cardiac defects observed in StarD7^−/−^ mice could be due, at least in part, to a decreased Cx43 expression. Furthermore, several reports clearly indicate that the development and function of the female reproductive tissues are regulated by connexins [54, 55]. Indeed, recurrent early pregnancy loss risk was linked with a decreased Cx43 transcript and protein expression in trophoblast cells [56, 57], highlighting the necessity to explore the StarD7 role in placental disorders.

**Fig 7:**
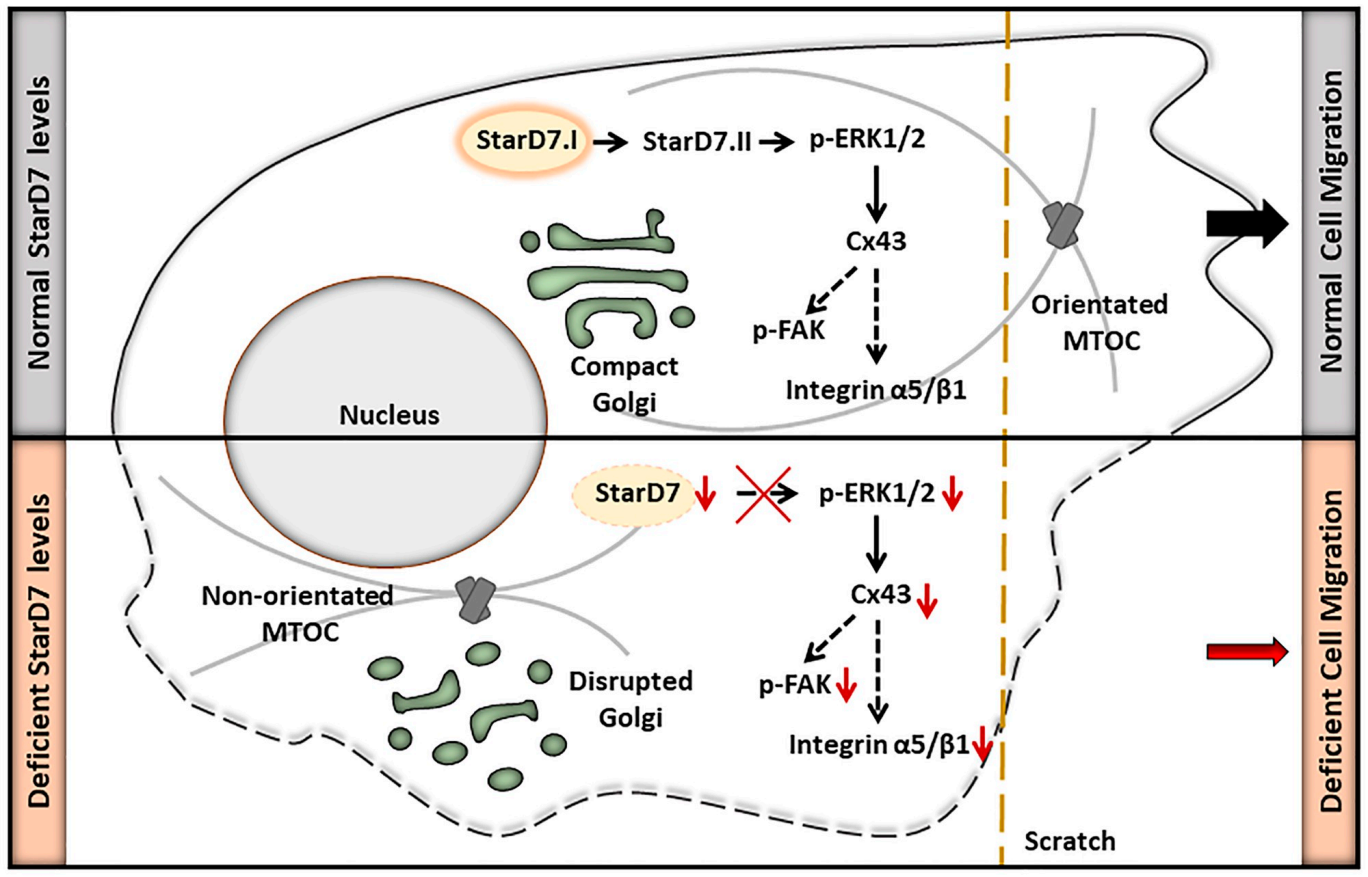
StarD7 deficiency impairs cell migration through a reduction in p-ERK1/2, Cx43, integrin α5/β1, p-FAK protein levels. It causes a disruption of the Golgi apparatus and a reduced competence to reorient the microtubule-organizing center (MTOC) during cell migration.

Collectively, these findings reveal that StarD7 depletion causes a reduction in p-ERK1/2/Cx43 levels impacting on cell migration behavior as well as in the expression of other proteins involved in this process, revealing a novel role for StarD7.II isoform (Fig 7).

## Supporting information

S1 Fig siRNA-mediated StarD7 silencing impairs MTOC reorientation and disrupts the Golgi apparatus. (A) The monolayer of siC or siD7 cells was wounded and 6 hours later cells were stained with anti-γ-tubulin (red) to detect MTOC (arrows). The boxed regions are enlarged in the right panels. The nuclei were labeled with Hoechst (blue), merged images are shown on the left. The images were recorded by fluorescence microscopy and white lines indicate the wound edge. Scale bar = 10 μm (600x). Bar graph shows the percentage of siC and siD7 cells exhibiting oriented MTOC after wound from two independent experiments (mean ± SEM, *p< 0.05, Student’s t-test). (B, C) Golgi apparatus morphology was visualized by fluorescence microscopy in siC and siD7 cells using anti-GM130 or anti-Golgin 97 antibodies, respectively (red). The nuclei were labeled with Hoechst (blue). Scale bar = 30 μm (200x). The boxed regions are enlarged in the right panels. Bar graph shows the percentage of siC and siD7 cells with fragmented Golgi from two independent experiments (mean ± SEM, *p< 0.05, Student’s t-test).
(TIF)

S2 Fig StarD7 knockdown does not alter mitochondrial ROS levels nor the Δψm. (A) MitoSOX Red was used to measure mitochondrial ROS in shD7 and shC cells. NC: unlabeled shC cells. The Histogram in the top panel depicts one representative of three independent experiments and the bar graph in the bottom panel shows the mean fluorescence intensity (mean ± SEM) relative to control defined as 100%. Statistical significance was evaluated by one-sample t-test. (B) JC-1 iodide dye was used to measure Δψm in shD7 and shC cells by flow cytometry in three independent experiments. One representative experiment is shown. Negative (non-stained cells) and positive (cells treated with 50 μM CCCP) controls were included in each experiment and are shown in the top panels.
(TIF)

S1 Raw images Raw material. (PDF)

S1 Movie Exogenous Cx43 restores cell migration in StarD7-deficient cells. Individual-cell migration recording in wound healing assays performed in shC cells transfected with EV-GFP plasmid. Cells move towards the acellular space located to the left of the dashed line.
(AVI)

S2 Movie Exogenous Cx43 restores cell migration in StarD7-deficient cells. Individual-cell migration recording in wound healing assays performed in shD7 cells transfected with EV-GFP plasmid. Cells move towards the acellular space located to the left of the dashed line.
(AVI)

S3 Movie Exogenous Cx43 restores cell migration in StarD7-deficient cells. Individual-cell migration recording in wound healing assays performed in shD7 cells transfected with pCx43-GFP. Cells move towards the acellular space located to the left of the dashed line.
(AVI)

## Abbreviations

Cx43: connexin 43
ECM: extracellular matrix
EGF: epidermal growth factor
ERK1/2: Extracellular signal-regulated kinases 1/2
GJIC: Gap junction intercellular communication
MFI: mean fluorescent intensity
MTOC: microtubule-organizing center
qRT-PCR: quantitative reverse transcription-polymerase chain reaction
shC: scrambled shRNA control sequence
shD7: shRNA StarD7 sequence
shRNA: short hairpin RNA
siC: control siRNA
siD7: StarD7 siRNA
siRNA: small interfering RNA
StarD7: StAR-related lipid transfer (START) domain containing 7
