# Supplementary figures and images for: StarD7 deficiency hinders cell motility through p-ERK1/2/Cx43 reduction
Source: PLoS One. 2022 Dec 30;17(12):e0279912. doi: 10.1371/journal.pone.0279912 (PMC9803278; doi:10.1371/journal.pone.0279912)

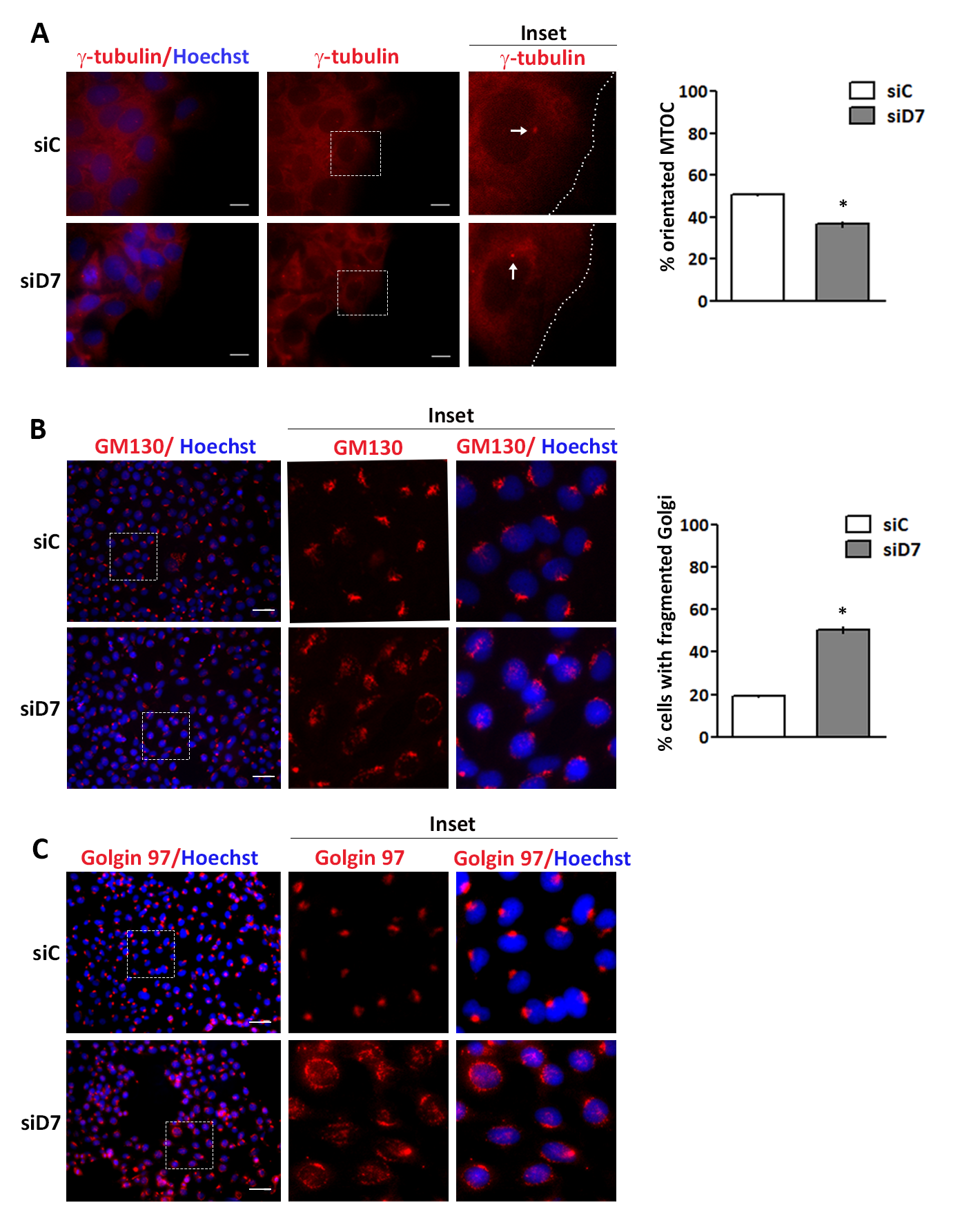

Supplement: S1 Fig — (A) The monolayer of siC or siD7 cells was wounded and 6 hours later cells were stained with anti-γ-tubulin (red) to detect MTOC (arrows). The boxed regions are enlarged in the right panels. The nuclei were labeled with Hoechst (blue), merged images are shown on the left. The images were recorded by fluorescence microscopy and white lines indicate the wound edge. Scale bar = 10 μm (600x). Bar graph shows the percentage of siC and siD7 cells exhibiting oriented MTOC after wound from two independent experiments (mean ± SEM, *p< 0.05, Student’s t-test). (B, C) Golgi apparatus morphology was visualized by fluorescence microscopy in siC and siD7 cells using anti-GM130 or anti-Golgin 97 antibodies, respectively (red). The nuclei were labeled with Hoechst (blue). Scale bar = 30 μm (200x). The boxed regions are enlarged in the right panels. Bar graph shows the percentage of siC and siD7 cells with fragmented Golgi from two independent experiments (mean ± SEM, *p< 0.05, Student’s t-test). (TIF) [file pone.0279912.s001.tif]

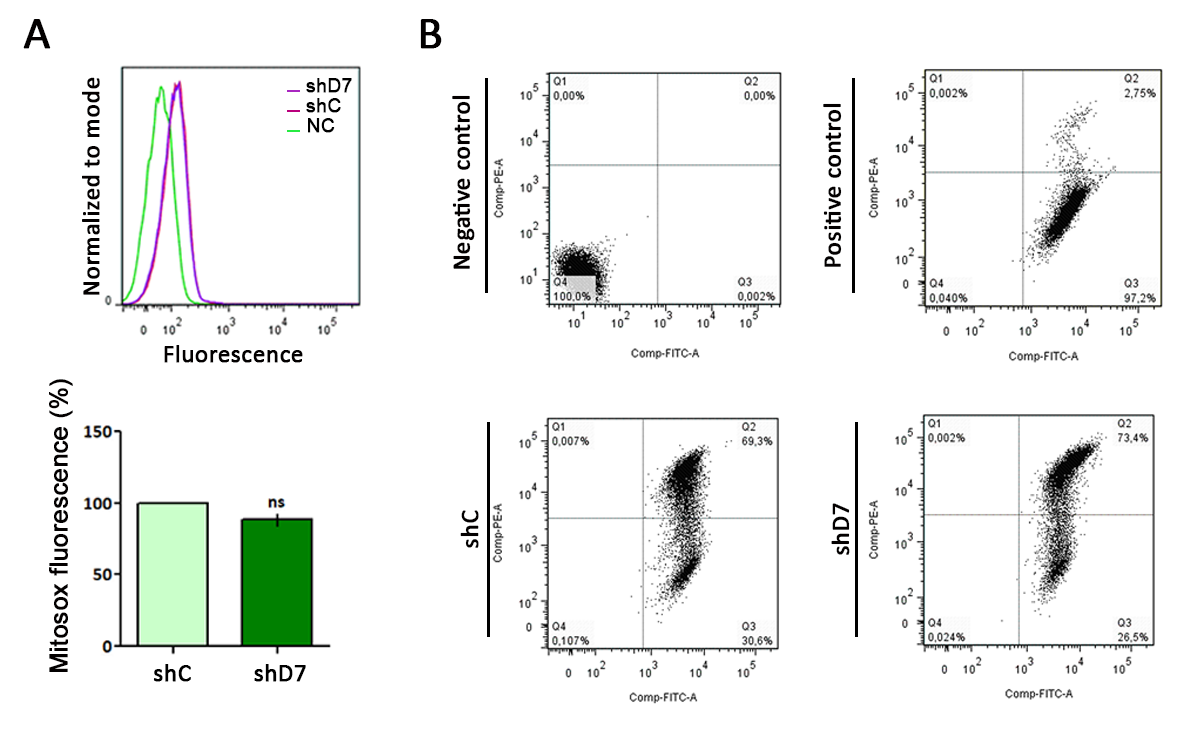

Supplement: S2 Fig — (A) MitoSOX Red was used to measure mitochondrial ROS in shD7 and shC cells. NC: unlabeled shC cells. The Histogram in the top panel depicts one representative of three independent experiments and the bar graph in the bottom panel shows the mean fluorescence intensity (mean ± SEM) relative to control defined as 100%. Statistical significance was evaluated by one-sample t-test. (B) JC-1 iodide dye was used to measure Δψm in shD7 and shC cells by flow cytometry in three independent experiments. One representative experiment is shown. Negative (non-stained cells) and positive (cells treated with 50 μM CCCP) controls were included in each experiment and are shown in the top panels. (TIF) [file pone.0279912.s002.tif]
