# Supplementary material for: StarD7 deficiency hinders cell motility through p-ERK1/2/Cx43 reduction
Source: PLoS One. 2022 Dec 30;17(12):e0279912. doi: 10.1371/journal.pone.0279912 (PMC9803278; doi:10.1371/journal.pone.0279912)
Supplement: S1 Raw images — (PDF) [file pone.0279912.s003.pdf]

Fig. 1

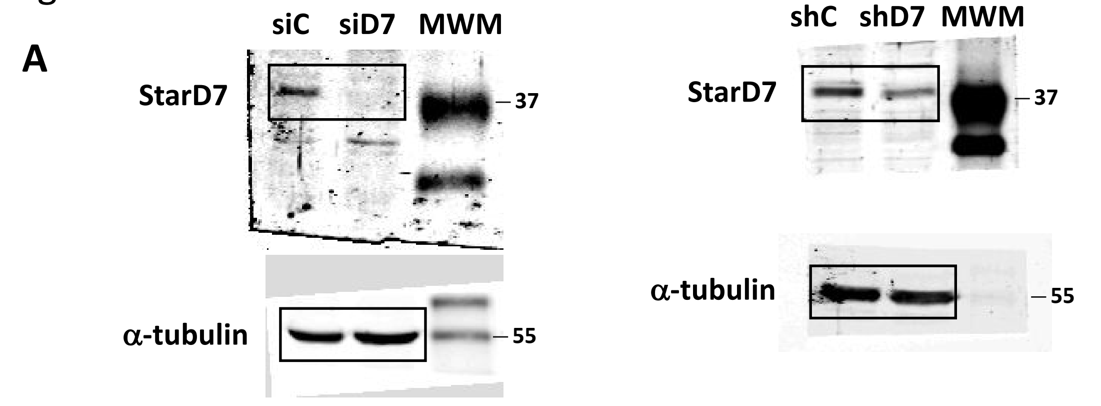

Fig. 2

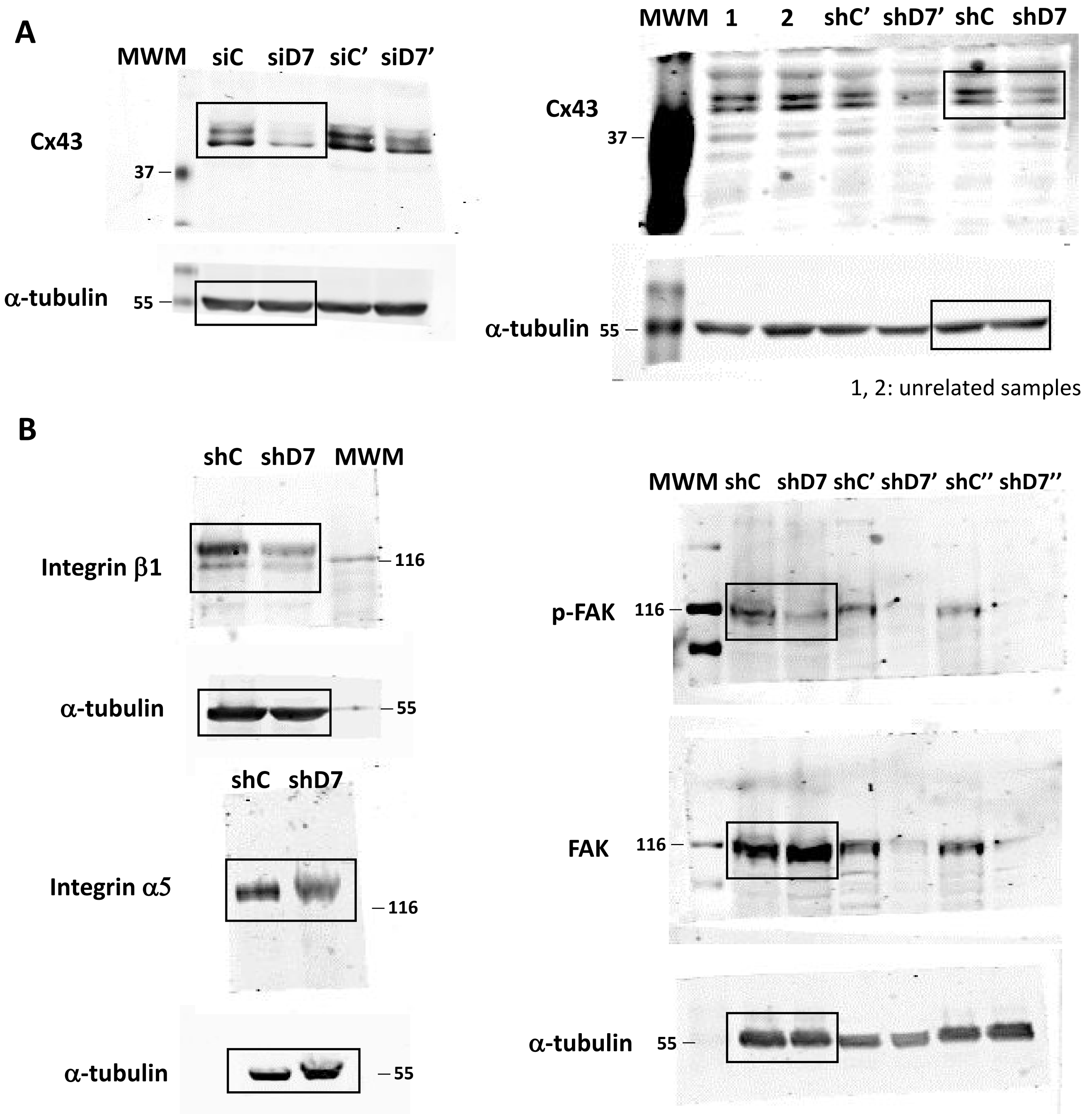

MWM: molecular weight marker

Fig. 3

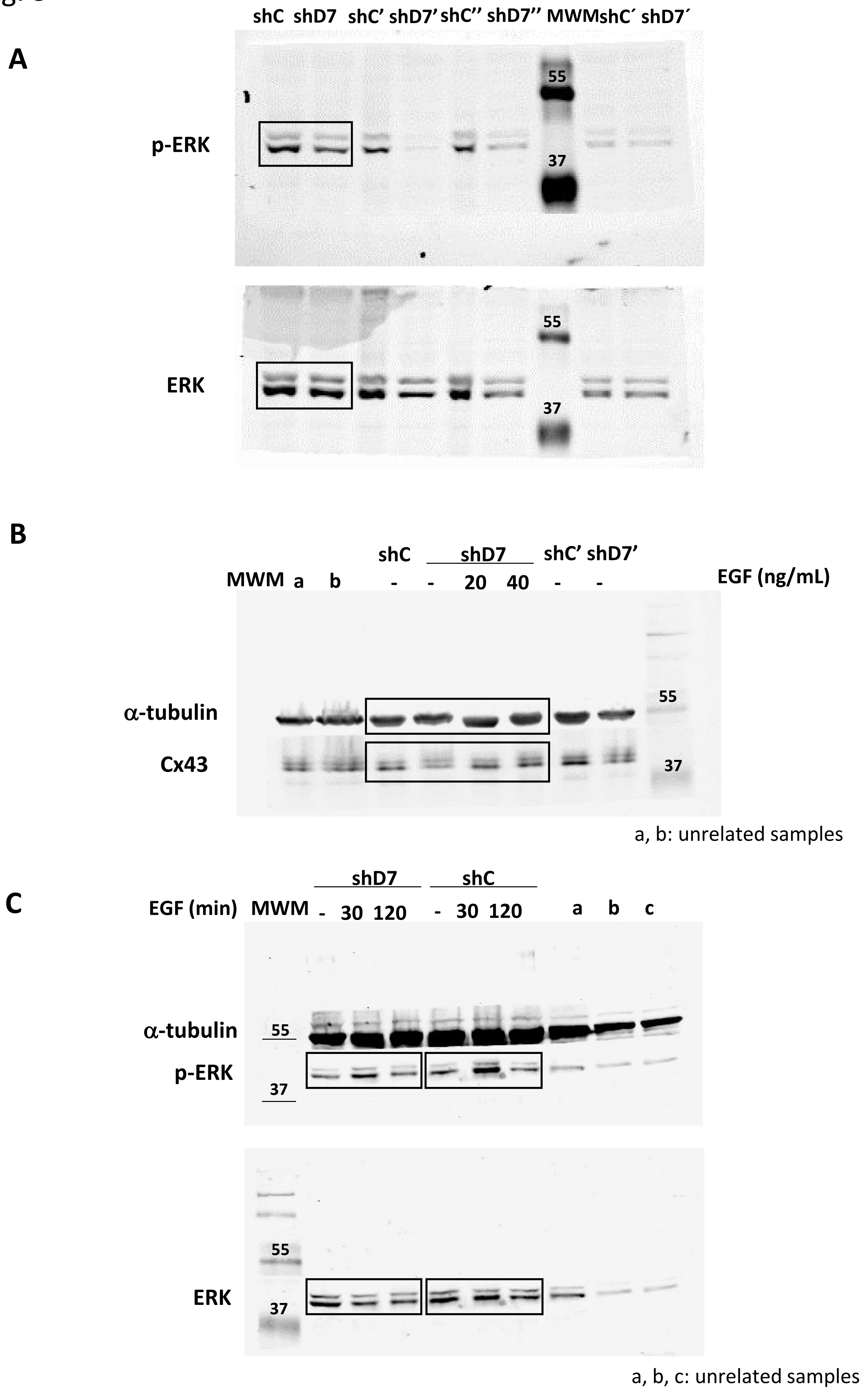

MWM: molecular weight marker

Fig. 5

C

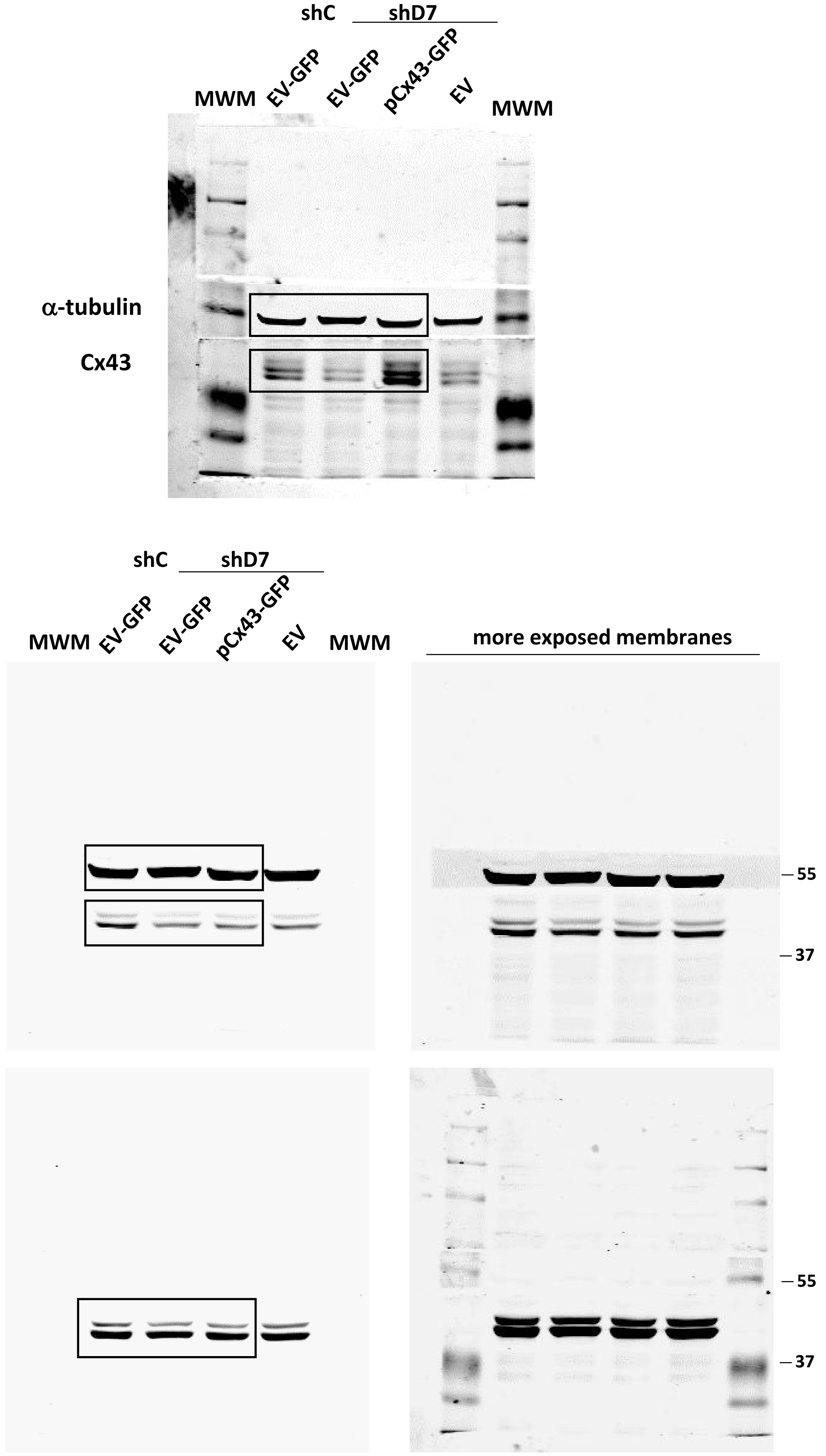

MWM: molecular weight marker

Fig. 6

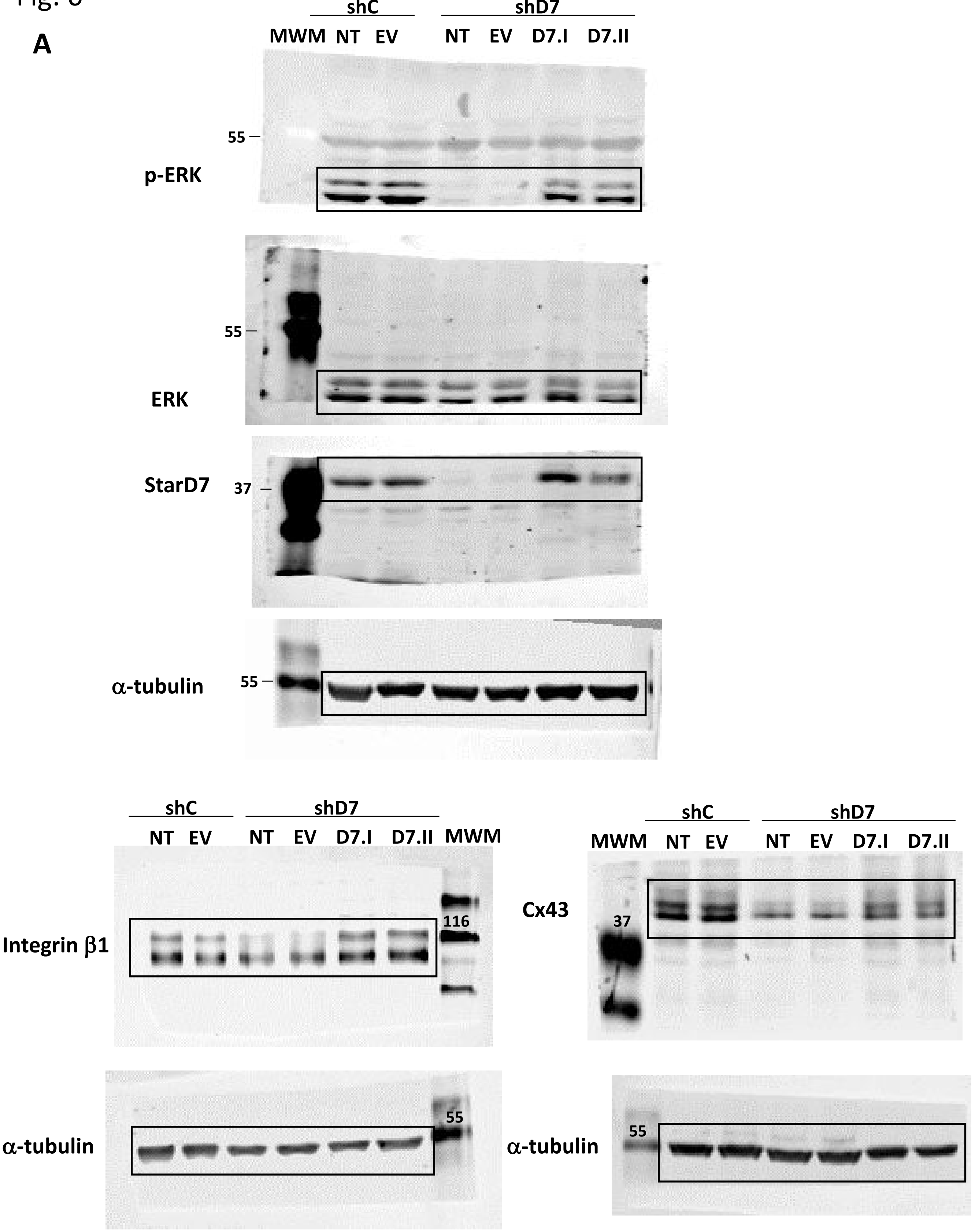

MWM: molecular weight marker
NT: not transfected
EV: empty vector
